# Supplementary material for: Splice-Junction-Based Mapping of Alternative Isoforms in the Human Proteome
Source: Cell Rep. Author manuscript; Available in PMC 2020 Jan 15. (PMC6961840; doi:10.1016/j.celrep.2019.11.026)
Supplement: 2 [file NIHMS1546469-supplement-2.zip › DF1/PXD009737/TestisTrypsin/TestisTrypsin_28_SYPL1_ETSLHSPSNTSAPHSQGGIPPPTGL.pdf]

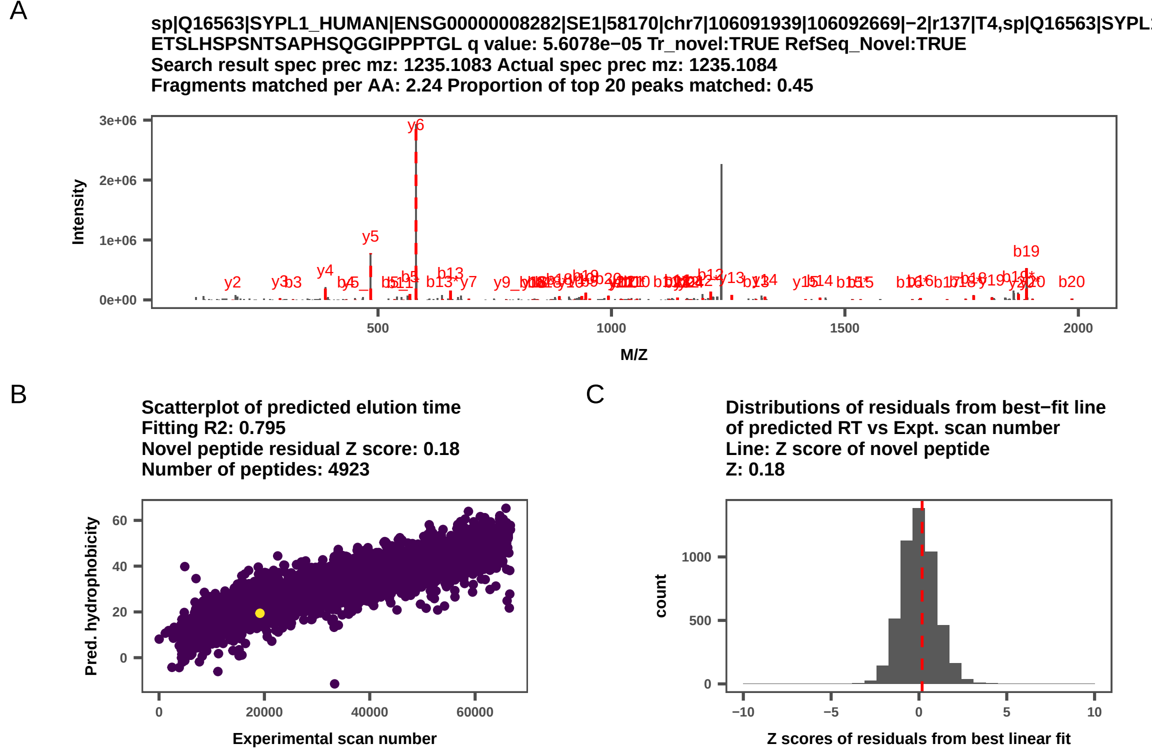
A
sp|Q16563|SYPL1_HUMAN|ENSG00000008282|SE1|58170|chr7|106091939|106092669|−2|r137|T4,sp|Q16563|SYPL
ETSLHSPSNTSAPHSQGGIPPPTGL q value: 5.6078e−05 Tr_novel:TRUE RefSeq_Novel:TRUE
Search result spec prec mz: 1235.1083 Actual spec prec mz: 1235.1084
Fragments matched per AA: 2.24 Proportion of top 20 peaks matched: 0.45
Intensity
3e+06
2e+06
1e+06
0e+00
y6
y5
y4
y2
y3
b3
b13
y7
b19
b20
500
1000
1500
2000
M/Z
B
Scatterplot of predicted elution time
Fitting R2: 0.795
Novel peptide residual Z score: 0.18
Number of peptides: 4923
Pred. hydrophobicity
60
40
20
0
0
20000
40000
60000
Experimental scan number
C
Distributions of residuals from best−fit line
of predicted RT vs Expt. scan number
Line: Z score of novel peptide
Z: 0.18
count
1000
500
0
−10
−5
0
5
10
Z scores of residuals from best linear fit
